# Robotic pancreatoduodenectomy for a giant duodenal leiomyoma: A case report and literature review

**DOI:** 10.1016/j.ijscr.2025.111546

**Published:** 2025-06-21

**Authors:** Susumu Doita, Kosei Takagi, Motohiko Yamada, Kazuya Yasui, Tomokazu Fuji, Toshiyoshi Fujiwara

**Affiliations:** Department of Gastroenterological Surgery, Okayama University Graduate School of Medicine, Dentistry, and Pharmaceutical Sciences, Okayama, Japan

**Keywords:** Duodenal leiomyomas, Robotic surgery, Pancreatoduodenectomy

## Abstract

**Introduction:**

Duodenal leiomyomas are rare mesenchymal tumors. To date, several studies have reported on the safety and feasibility of surgical intervention for duodenal leiomyomas. However, minimally invasive surgery has rarely been performed in cases with duodenal leiomyomas. Herein, we present a case of a giant duodenal leiomyoma successfully treated with robotic pancreatoduodenectomy (RPD).

**Presentation of case:**

A 74-year-old man was referred to our hospital with a 6.5 cm duodenal tumor accompanied by gastrointestinal bleeding. The tumor was located in the second portion of the duodenum. Considering the tumor size and location, RPD was performed. Using the mesenteric Kocker maneuver, the posterior side of the duodenum was safely dissected, and the tumor was resected. The operative time was 373 min, with an estimated blood loss of 10 mL. The patient was followed up for 7 months with no recurrence.

**Discussion:**

To the best of our knowledge, this is the first to highlight the clinicopathological findings of a patient with duodenal leiomyoma undergoing RPD. To date, there have been 19 cases, including our case, reporting surgically treated duodenal leiomyoma. Treatment strategies should be decided depending on tumor characteristics, including the size, location, and histology of the tumor.

**Conclusion:**

We present a rare case of a giant duodenal leiomyoma that was successfully treated with RPD. Minimally invasive surgery can be safe and an alternative for the treatment of large duodenal tumors.

## Highlights

• Duodenal leiomyomas are rare mesenchymal tumors.
• We present a rare case of a giant duodenal leiomyoma that was successfully treated with robotic pancreatoduodenectomy.
• Minimally invasive surgery can be safe and an alternative for the treatment of large duodenal tumors.

## 1 Introduction

Leiomyomas are the common benign mesenchymal tumors of the upper gastrointestinal tract [1]. Since the commonest site of its occurrence is the stomach, duodenal leiomyomas are rare. The current treatment strategies for benign duodenal tumors include endoscopic or surgical resections; however, surgical intervention, including local resection and pancreatoduodenectomy (PD), should be considered in cases with large tumors (>2 cm) [2]. Although conventional open PD is associated with high morbidities [3], minimally invasive PD could be an alternative to overcome the shortcomings of open PD. Despite the growing evidence of robotic PD (RPD), few studies have reported on cases with duodenal leiomyoma undergoing RPD.

In this report, we present a rare case of a giant duodenal leiomyoma successfully treated with RPD. The work has been reported in line with the SCARE criteria [4].

## 2 Case presentation

**Fig. 1:**
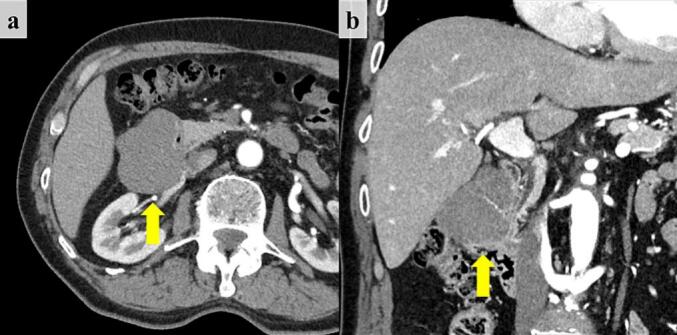
Abdominal computed tomography image. (a) Axial view: The 6.5 cm well-defined low-enhanced mass (arrow) is located in the second portion of the duodenum. (b) Coronal view.

A 74-year-old man was referred to our hospital with a duodenal tumor accompanied by gastrointestinal bleeding. Laboratory tests detected only anemia (hemoglobin level, 12.0 g/dL), but no elevated tumor marker. Abdominal contrast-enhanced computed tomography revealed a 6.5 × 5.0 cm well-defined low-enhanced mass in the duodenum (Fig. 1). An upper gastrointestinal endoscopy showed a lateral three quarters circumferential submucosal tumor in the second part of the duodenum. An endoscopic biopsy revealed the suspicion of a duodenal leiomyoma. Considering the tumor size and location, a surgical intervention with RPD using the da Vinci Xi system was performed instead of an endoscopic intervention.

**Fig. 2:**
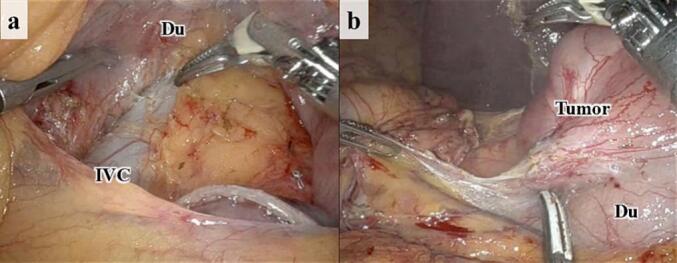
Intraoperative findings. (a) Caudal view through the mesenteric route. Using the mesenteric Kocker maneuver, the posterior side of the duodenum was safely dissected. (b) Ventral view. *Du* duodenum, *IVC* inferior vena cava.

Details of our surgical protocol for RPD have been previously reported [5]. Intraoperative findings showed no metastasis or peritoneal dissemination. Initially, the mesenteric Kocker maneuver was performed to mobilize the pancreatic head and the duodenum tumor, showing no tumor invasion (Fig. 2) [6]. Following the division of the jejunum and the stomach, the pancreas was transected on the superior mesenteric vein. The hepatoduodenal ligament and uncinate process were dissected to remove the specimen. The reconstruction included pancreaticojejunostomy using the modified Blumgart method, gepaticojejunostomy, and gastrojejunostomy [5]. The operative time was 373 min, with an estimated blood loss of 10 mL. The patient was discharged on postoperative day 10, and was followed up for 7 months with an uneventful clinical course.

**Fig. 3:**
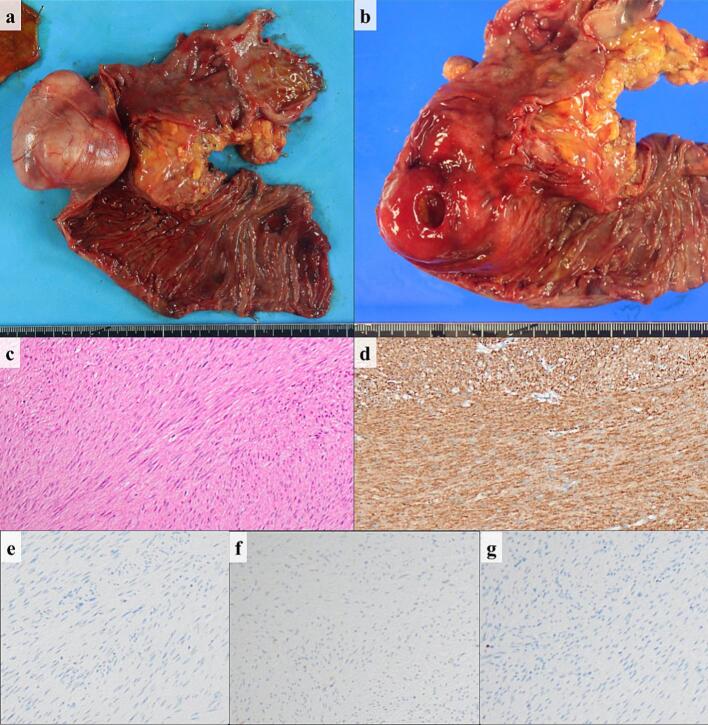
Pathological findings. (a) Gross examination showing a 6.5 cm well-circumscribed mass in the duodenum; (b) A central ulceration in the tumor; (c) Microscopic examination showing the proliferation of spindle-shaped cells with poor atypia in an intricate manner (Hematoxylin and Eosin staining); Immunohistochemically, the tumor cells are positive for α-SMA (d) and negative for S-100 (e) and c-kit (f); (g) The Ki-67 index was <1 %.

The gross examination revealed a 6.5 cm, soft, well-circumscribed, submucosal mass with a central ulceration in the duodenum (Fig. 3). Histological findings showed the proliferation of spindle-shaped cells with poor atypia intricately. Immunohistochemically, the tumor cells were positive for α-smooth muscle actin and negative for c-kit and S-100. The Ki-67 index was <1 %. Accordingly, the tumor was diagnosed as duodenal leiomyoma.

## 3 Discussion

The present study demonstrated a rare case of a giant duodenal leiomyoma successfully treated with RPD. To the best of our knowledge, this is the first to highlight the clinicopathological findings of a patient with duodenal leiomyoma undergoing RPD. Moreover, this study reviewed clinicopathological features in reported cases of duodenal leiomyoma.

To date, there have been 19 cases, including our case, reporting surgically treated duodenal leiomyoma (Table 1) [[7], [8], [9], [10], [11], [12], [13]]. The mean tumor size was 3.8 cm (range 1–12 cm), and there were four cases with giant tumors (>5 cm). Most of the patients had symptoms such as anemia and gastrointestinal bleeding. The common surgical approach was open surgery, whereas laparoscopic surgery was used in two patients and robotic surgery in two patients. Regarding the type of procedures, duodenectomy with or without reconstruction was performed in 15 patients, and four patients underwent PD. In cases with partial sleeve duodenectomy, subsequent reconstruction including duodenojejunostomy and Billroth II gastrojejunostomy were performed [9]. Good long-term outcomes after surgical resection have been reported with no recurrence.

**Table 1:** Clinicopathological features in reported cases of surgically treated duodenal leiomyoma.

Author	Year	Age (year)/sex	Symptom	Tumor size (cm)	Tumor location	Procedure	Outcome
Chong et al. [7]	2000	26/F	NA	NA	NA	Laparoscopic local excision	NA
Rice et al. [8]	2001	70/NA	Abdominal pain	2.5	3rd/4th	Segmental resection followed by primary anastomosis	12.5 years, no recurrence
		71/NA	Anemia	3.0	2nd	Segmental resection followed by primary anastomosis	1.0 years, no recurrence
		71/NA	Abdominal pain	1.1	2nd	PD	6.0 years, no recurrence
		69/NA	Abdominal pain/weight loss	1.0	1st	PD	5.1 years, no recurrence
		50/NA	Anemia	2.5	2nd	Local excision	17.3 years, no recurrence
		70/NA	Anemia	2.7	4th	Local excision	NA
		30/NA	Dyspepsia/anemia	3.5	2nd	Local excision	8.4 years, no recurrence
		69/NA	Anemia	3.2	2nd	Local excision	9.0 years, no recurrence
		48/NA	Melena	1.0	2nd	Local excision	NA
		73/NA	Anemia	1.0	2nd	Local excision	3.4 years, no recurrence
		20/NA	Anemia	3.6	2nd	Local excision	14.5 years, no recurrence
		54/NA	Dyspepsia/anemia	2.8	3rd/4th	Local excision	NA
Stauffer et al. [9]	2013	79/F	NA	2.4	3rd/4th	Laparoscopic distal duodenectomy followed by primary anastomosis	NA
Downs-Canner et al. [10]	2015	NA/NA	NA	NA	NA	Robotic duodenal resection	NA
Chai et al. [11]	2016	65/M	Abdominal pain/anemia	7.0	2nd	PD	NA
Nonoyama et al. [12]	2018	44/F	Anemia	12.0	3rd	Segmental resection followed by primary anastomosis	2.5 years, no recurrence
Sy et al. [13]	2018	69/M	GIB	9.0	2nd	Local excision	NA
Our case		74/M	GIB	6.5	2nd	RPD	7 months, no recurrence

*M* male, *F* female, *GIB* gastrointestinal bleeding, *IVR* interventional radiology, *PD* pancreatoduodenectomy, *RPD* robotic pancreatoduodenectomy, *NA* not available.

Treatment strategies should be decided depending on tumor characteristics, including the size, location, and histology of the tumor. A small asymptomatic tumor may be closely followed up. However, symptomatic or larger tumor should be treated. Although endoscopic intervention may be associated with reduced trauma and faster recovery, it is more challenging with high risks of complications, including perforation and bleeding [14]. Therefore, larger lesions and those that involve the submucosa should be treated with surgical intervention [7]. When performing surgical intervention, local excision without reconstruction may be the first option. Next, duodenal segmental resection followed by primary anastomosis can be considered. Various types of reconstruction should be performed depending on tumor location [9]. Finally, the most commonly practiced approach using PD should be selected.

In the present case, a symptomatic tumor with gastrointestinal bleeding was considered as an indication of therapeutic intervention. Since the tumor was lateral three quarters circumferential in the duodenum contacting the pancreas, local resection was difficult. Given the tumor size and location, the Whipple procedure was required instead of endoscopic or surgical local resection. As the Whipple procedure may be too aggressive for benign duodenal tumors, we selected a minimally invasive approach using robotic surgery. Regarding surgical techniques, the mesenteric Kocker maneuver can help to dissect behind the large duodenal tumor.

## 4 Conclusions

This study presents a rare case of a giant duodenal leiomyoma that was successfully treated with RPD. Minimally invasive surgery can be an alternative for the treatment of large duodenal tumors.

## Consent

Written informed consent was obtained from the patient for publication and any accompanying images. A copy of the written consent is available for review by the Editor-in-Chief of this journal on request.

## Ethical approval

We certify that this kind of manuscript does not require ethical approval (exemption) by the Ethical Committee of our institution (Okayama University Hospital).

## Guarantor

Kosei Takagi

## Research registration number

Not applicable.

## Funding

There are no sources of funding.

## Author contribution

Susumu Doita: Conceptualization, Patient management.

Kosei Takagi: Data curation, Writing- Original draft preparation.

Motohiko Yamada: Conceptualization, Patient management.

Kazuya Yasui: Conceptualization, Patient management.

Tomokazu Fuji: Patient management, Writing- Reviewing and Editing.

Toshiyoshi Fujiwara: Supervision, Writing- Reviewing and Editing.

## Conflict of interest statement

The authors have no conflicts of interest to declare.
